# Retrospective analysis of acute admissions for interstitial lung disease suggests a complex, multifactorial association between social deprivation and 90-day all-cause mortality: data from the North West of England

**DOI:** 10.1136/bmjresp-2025-003944

**Published:** 2026-07-08

**Authors:** Laura J White, Jonathon Shaw, Bethan Powell, Nyan M Kyi, Alicia Sou, Gareth Edward Hughes, Dilanka Tilakaratne, Conal Hayton, Trishala Raj, Vi Truong, Nashwah Ismail, Nawat Khanijoun, Rebecca Huang, Emma Hardy, Mahzaib Babar, Naayaab Khan, Martin Regan, Oby Okpala, Ragavilasini Suresh, Jerome Mcintosh, Amsal Amjad, Mahum Sohail, Zainab Aslam, Amy Gadoud, Timothy Gatheral, Georges Ng Man Kwong

**Affiliations:** 1 Lancaster Medical School, Lancaster University, Lancaster, England, UK; 2 Respiratory Medicine, University Hospitals of Morecambe Bay NHS Foundation Trust, Kendal, England, UK; 3 Department of Respiratory Medicine, Stockport NHS Foundation Trust, Stockport, UK; 4 Department of Respiratory Medicine, Blackpool Teaching Hospitals NHS Foundation Trust, Blackpool, UK; 5 Department of Respiratory Medicine, Northern Care Alliance NHS Foundation Trust, Salford, England, UK; 6 Department of Respiratory Medicine, Bolton NHS Foundation Trust, Bolton, UK; 7 Division of Infection, Immunity & Respiratory Medicine, The University of Manchester, Manchester, UK; 8 School of Health, Science and Society, University of Greater Manchester, Bolton, UK; 9 Department of Respiratory Medicine, Manchester University NHS Foundation Trust, Manchester, England, UK; 10 School of Medical Sciences, The University of Manchester, Manchester, England, UK; 11 Department of Respiratory Medicine, Tameside and Glossop Integrated Care NHS Foundation Trust, Ashton-under-Lyne, England, UK; 12 Pears Cumbria School of Medicine, Imperial College London, Carlisle, UK

**Keywords:** Clinical Epidemiology, Interstitial Fibrosis, Idiopathic Pulmonary Fibrosis, Patient Outcome Assessment

## Abstract

**Background:**

Social deprivation impacts chronic disease and acute admission outcomes. In interstitial lung disease (ILD), prior British Thoracic Society registry data for idiopathic pulmonary fibrosis have shown high deprivation was associated with poorer long-term outcomes. However, its impact on acute admissions in ILD is not known.

**Methods:**

We undertook a multicentre, retrospective study of ILD-related admissions between 1 January 2017 and 31 December 2019 across 11 hospitals in the North West of England, using available real-world data. We determined social deprivation geographically by the 2019 English Indices of Deprivation deciles. The primary outcome was 90-day all-cause mortality.

**Results:**

999 admissions met the inclusion criteria. 327/999 (32.7%) of admissions came from individuals geographically in the most deprived 20%. Across 999 admissions, in unadjusted survival analysis, we observed a non-linear relationship between deprivation and 90-day all-cause mortality. In complete case multivariate modelling, deprivation demonstrated borderline significant association with all-cause mortality (HR 1.038, 95% CI 1.00 to 1.077, p=0.050). However, this effect was lost in pooled analysis using multiple imputation (HR 1.001, 95% CI 0.971 to 1.033, p=0.924). Male sex and pre-admission long-term oxygen were consistently associated with increased 90-day all-cause mortality across both models. Lower transfer factor for carbon monoxide values were significantly associated with increased 90-day mortality in pooled analysis.

**Conclusion:**

We observe a high burden of acute ILD-related hospital admission among the most deprived 20%, suggesting geographical deprivation may impact acute healthcare seeking behaviours. Once admitted, the impact of deprivation appears more complex and multifactorial. Further studies which assess geographical and individual-level deprivation are needed to validate our findings.

**WHAT IS ALREADY KNOWN ON THE TOPIC**

- The British Thoracic Society idiopathic pulmonary fibrosis registry has previously demonstrated that higher social deprivation is associated with worse long-term outcomes. In other respiratory diseases, social deprivation impacts acute admission patterns and outcomes.

**WHAT THIS STUDY ADDS**

- To the best of our knowledge, this is the first study examining the relationship between social deprivation and acute interstitial lung disease (ILD)-related admission outcomes. This study demonstrates high acute admission burden from the geographically most deprived 20%. Once admitted, the association between geographical social deprivation and mortality outcomes appears complex and multifactorial in our modelling.

**HOW THIS MAY AFFECT RESEARCH, PRACTICE OR POLICY**

- This study highlights the acute admission burden from highly deprived communities and the need for additional research to further understand the individual-level and geographical-level deprivation patients with ILD experience. We suggest the need for community outreach to build trust with deprived communities, alongside increasing awareness among patients, caregivers and primary care physicians in such communities. Deprivation must remain an important consideration in any new service or intervention to prevent worsening of health inequalities.

## Introduction

 Social determinants of health (SDH) describe the non-medical components of health which influence disease outcomes.^1^ Social deprivation is a complex entity which describes one component of this. According to the UK Government, deprivation is defined as an individual who lacks “any kind of resources”, whereas poverty refers to the specific lack of financial resources to meet needs.^2^ Inequalities in health and outcomes in chronic disease are closely associated with SDH. The WHO estimates non-medical factors contribute to between 30% and 55% of disease outcomes.^3^

In the 2019 English indices of deprivation, seven domains determine an Index of Multiple Deprivation (IMD) for each small locality, related to postcode. These domains include weighted averages of income (22.5%), employment (22.5%), education (13.5%), health and disability (13.5%), crime (9.3%), barriers to housing and services (9.3%), and living environment (9.3%).^4^ Local authorities are subsequently ranked and split into 10, determining the 10 deciles of deprivation. Importantly, these deciles reflect average geographical deprivation—rather than individual-level deprivation statistics.

Interstitial lung diseases (ILD) represent a heterogenous group of disorders affecting the lung parenchyma. Idiopathic pulmonary fibrosis (IPF) is the archetypal progressive fibrotic form of ILD. Data from the British Thoracic Society (BTS) IPF registry found those from the most deprived social backgrounds had more severe disease at diagnosis and worse long-term outcomes.^5^ In ILD more generally, living and working conditions were most frequently associated with health inequalities—and thus overall patient outcomes.^3^ The North West of England has the highest rate of deprivation in the UK, with 45% of districts ranked in the most deprived decile located in the region.^6^ Given the evidence of health inequality in chronic disease, combined with high levels of social deprivation in the North West, we sought to determine if social deprivation impacts 90-day mortality outcomes from acute ILD-related hospital admissions in this region.

## Methods

### Study setting and population

We performed a multicentre retrospective observational study of acute ILD-related hospital admissions in adults ≥18 years old. The term ‘acute ILD-related hospital admissions’ was used to encompass all acute admissions deemed associated with an underlying ILD diagnosis, inclusive of acute ILD presentation, acute exacerbation of interstitial lung disease (AEILD) and progression of disease. Admissions were identified using International Classification of Disease Version 10 (ICD-10) coding between 1 January 2017 and 31 December 2019 from 11 National Health Service (NHS) secondary and tertiary hospitals in the North West of England (online supplemental figure 1). ICD-10 codes of B22.1, D86.0, D86.2, J67.0-67.9, J70.2-70.4, J84.1, J84.8 and J84.9 within the primary diagnosis of admission event triggered review for inclusion. Reasons for exclusion are summarised in online supplemental figure 1, including any prior documented dissent for use of data in research.

Baseline admission and patient characteristics data were sourced from coding and manual searching of medical records. Full postcodes were converted to deprivation deciles (DD) as per the 2019 UK Government English Indices of Deprivation data, with a DD of 1 representing the most deprived 10% and a DD of 10 the least deprived 10%.^2^ Admission events meeting criteria were subsequently grouped into quintiles, with quintile 1 representing the least deprived (DDs 9 and 10) and quintile 5 representing the most deprived (DDs 1 and 2). Primary outcome was number of days from start of admission to death.

### Patient and public involvement

Following the development of the study protocol, the concept and planned design was presented to a group of expert patients, the Research Champions through Action for Pulmonary Fibrosis, prior to Integrated Research Application System submission. They confirmed acceptability of the proposed design and assisted with dissemination strategy, including radio and newsletter appearances following publication. Given the retrospective data design of the study, no further patient or public involvement was undertaken.

The Action for Pulmonary Fibrosis Research Champions are a group of expert patients involved in charitable and research activities related to ILD. While it was beneficial to discuss the proposed study with this group, further details on their socioeconomic status (SES) were not requested nor recorded. In retrospect, given the significant proportion of acute admissions from the most deprived 20% of the population, this would have been beneficial to understanding the relevance for the population these results impact. Future studies should seek input from deprived communities, perhaps away from structured support group settings.

### Data collection and variables

All data were sourced from coding and manual searching of medical records by site-specific clinicians. All admissions meeting inclusion criteria underwent manual review of medical notes. Data, where available, were collected on patient demographics, co-morbidities (converted to Charlson Comorbidity Index (CCI)^7^) and pre-existing ILD diagnoses. Underlying ILD diagnosis was obtained from manual searching of medical records, using multidisciplinary team (MDT) documentation, radiology and clinic letters where available. During data collection, we observed a group of patients without an MDT-confirmed diagnosis, but in whom evidence of fibrotic ILD was present. This group was labelled ‘pulmonary fibrosis as a diagnostic label’ (PFD), reflecting real-world access to ILD MDT diagnosis. Additional ILD-specific data including antifibrotic use, pre-admission oxygen use and pre-admission lung function testing were recorded where available.

Admission-relevant data included mortality outcomes and at-admission biochemical investigations (white cell count and differential, C-reactive protein (CRP)). As part of the manual search of medical records, site-specific clinicians assessed the admission reason using information available from the clinical narrative, biochemical and radiological investigations. For the purposes of this study, an AEILD event was defined clinically by <30-day clinical deterioration, not secondary to cardiac failure, thromboembolic disease (TED) or pneumothorax event. Whether AEILD criteria were met was at the discretion of the site-specific clinician. If not met, they were deemed to have an ‘other’ ILD-related hospital admission. Other ILD-related admission reasons included disease progression, pulmonary embolism, pulmonary hypertension and pneumothorax but were not recorded as discrete variables. Both types of admissions are grouped together under the umbrella term ‘acute ILD-related hospital admissions’.

### Statistical analysis

Statistical analysis was undertaken using SPSS v31.0 and Python v3.9.7. Continuous data were assessed for normality with Shapiro-Wilk test. Statistical comparison between quintiles was undertaken by one-way analysis of variance or Kruskal-Wallis H, χ² or Fisher’s exact test as appropriate, with post-hoc analysis using Dunn’s test for continuous variables and standardised residuals for categorical variables, with Bonferroni correction. Time-to-event survival analysis was undertaken by Kaplan-Meier with log-rank comparison.

HRs were initially estimated using a complete case multivariate Cox proportional hazards model with the Enter method—whereby all variables are entered into the model simultaneously. Variable selection for the model was based on clinical relevance and prior evidence in mortality outcomes, rather than data-driven stepwise methods, to minimise model overfitting. Demographic characteristics (including age, gender, ethnicity) were included to assess the impact of non-modifiable risk factors, while clinical and inflammatory variables were chosen for their known or hypothesised relevance to mortality risk in ILD populations.^8^,^10^ Variables with high proportions of missing data were not included (with a threshold of ≥15% missing data used) for complete case analysis. As a result, forced vital capacity (FVC, 387/999 or 38.7% missing), transfer factor for carbon monoxide (TLCO, 596/999 or 59.6% missing) and oxygen (L/min) at admission (679/999, or 67.9% missing) were not included in the model due to high levels of data missingness (online supplemental table 3). While rates of antifibrotic medication use were statistically significantly different between the quintiles (p=0.002; table 1), there was an overall low rate of antifibrotic use. As such, this was not included in the multivariate modelling due to the risk of model over-fitting and inaccurate estimates of mortality association due to small numbers. The proportional hazards assumption was checked using Schoenfeld residuals.

**Table 1:** Full descriptive baseline characteristics of each deprivation quintile, based on the 2019 English Indices of Deprivation

	Quintile 1 (DD 9 and 10) n=130	Quintile 2 (DD 7 and 8) n=175	Quintile 3 (DD 5 and 6) n=153	Quintile 4 (DD 3 and 4) n=214	Quintile 5 (DD 1 and 2) n=327	P value
Age (mean, SD)	76.62 (9.53)	76.06 (11.64)	74.64 (10.99)	75.73 (10.63)	70.21 (15.58)	**<0.0001**
Sex—male (number, %)	82 (63.08)	97 (55.43)	90 (58.82)	116 (54.21)	165 (50.46)	0.130
Ethnicity (number, %) Not stated White Asian Black	9 (6.92) 116 (89.23) 5 (3.85) 0 (0.00)	11 (6.29) 155 (88.57) 8 (4.57) 1 (0.57)	9 (5.88) 137 (89.54) 6 (3.92) 1 (0.65)	15 (7.01) 189 (88.32) 10 (4.67) 0 (0.00)	20 (6.12) 259 (79.20) 40 (12.23) 8 (2.45)	**0.0008**
CCI (mean, SD)	4.63 (1.95)	4.64 (1.93)	4.44 (1.95)	4.71 (1.91)	4.04 (2.04)	**0.002**
Smoking status (number, %) Not available Current Ex-smoker (>3 months) Never smoker	15 (11.54) 4 (3.08) 63 (48.46) 48 (36.92)	20 (11.43) 5 (2.86) 92 (52.57) 58 (33.14)	27 (17.65) 6 (3.92) 74 (48.37) 46 (30.07)	47 (21.96) 7 (3.27) 107 (50.00) 53 (24.77)	67 (20.49) 19 (5.81) 139 (42.51) 102 (31.19)	**0.043**
Pre-existing ILD diagnosis (number, %)	108 (83.08)	140 (80.00)	122 (79.74)	176 (82.24)	256 (78.29)	0.799
ILD subtype (number, %) Not stated IPF NSIP CTD-ILD HP Drug-related Industry-related Sarcoidosis PFD Unclassifiable Other	11 (8.46) 45 (34.62) 21 (16.15) 10 (7.69) 10 (7.69) 2 (1.54) 3 (2.31) 3 (2.31) 23 (17.69) 0 (0.00) 2 (1.54)	13 (7.43) 68 (38.86) 8 (4.57) 8 (4.57) 19 (10.86) 8 (4.57) 2 (1.14) 6 (3.43) 36 (20.57) 4 (2.29) 3 (1.71)	20 (13.07) 39 (25.49) 15 (9.80) 6 (3.92) 11 (7.19) 6 (3.92) 2 (1.31) 1 (0.65) 48 (31.37) 1 (0.65) 4 (2.61)	23 (10.75) 56 (26.17) 19 (8.88) 10 (4.67) 19 (8.88) 3 (1.40) 6 (2.80) 4 (1.87) 56 (26.17) 14 (6.54) 4 (1.87)	33 (10.09) 101 (30.89) 27 (8.26) 18 (5.50) 18 (5.50) 3 (0.92) 5 (1.53) 14 (4.28) 76 (23.24) 10 (3.06) 22 (6.73)	**<0.0001**
On antifibrotics (number, %)	8 (6.15)	35 (20.0)	17 (11.11)	19 (8.88)	44 (13.46)	**0.002**
Oxygen used pre-admission (number, %) Long-term Ambulatory None	26 (20.00) 11 (8.46) 93 (71.54)	42 (24.00) 9 (5.14) 124 (70.86)	43 (28.10) 9 (5.88) 101 (66.01)	59 (27.57) 22 (10.28) 133 (62.15)	96 (29.36) 30 (9.17) 201 (61.47)	0.205
FVC (L) (mean, SD)	2.19 (0.83)	2.09 (0.80)	2.18 (0.89)	2.13 (0.79)	2.04 (0.85)	0.590
TLCO (mm Hg) (mean, SD)	4.22 (3.08)	3.87 (2.11)	4.03 (2.03)	3.56 (1.65)	3.89 (2.12)	0.728
AEILD status (number, %) AEILD Other admission Not available	71 (54.62) 50 (38.46) 9 (6.92)	87 (49.71) 79 (45.14) 9 (5.14)	67 (43.79) 82 (53.59) 4 (2.61)	127 (59.35) 77 (35.98) 10 (4.67)	158 (48.32) 137 (41.90) 32 (9.79)	**0.003**
Oxygen (L/min) at admission (mean, SD)	2.94 (4.34)	3.21 (4.51)	3.17 (4.42)	3.62 (5.04)	2.37 (3.81)	0.765

Continuous variables were described using mean (SD) and subsequently tested for normality with the Shapiro-Wilk test. Quintiles were compared using ANOVA or Kruskal-Wallis, dependent on normality. Categorical variables were described using number frequency (percentage) and compared using χ² or Fisher’s exact test, dependent on expected values greater or less than five. A global comparative p value is reported.

Quintile 1 represents the least deprived 20%, equivalent to deprivation deciles 9 and 10, and quintile 5 the most deprived 20%, equivalent to deprivation deciles 1 and 2.

Additional note: post-hoc pairwise comparison analysis for age and CCI using Dunn’s test with Bonferroni correction available as online supplemental table 1. Post-hoc analysis for categorical variables using standardised residuals and pairwise comparison with Bonferroni correction is available as online supplemental table 2.

* Statistically significant values are marked in bold.

AEILD, acute exacerbation of interstitial lung disease; CCI, Charlson Comorbidity Index; CTD-ILD, connective tissue disease interstitial lung disease; DD, deprivation decile; FVC, forced vital capacity; HP, hypersensitivity pneumonitis; IPF, idiopathic pulmonary fibrosis; L, litres; mm Hg, millimetres of mercury; NSIP, non-specific interstitial pneumonia; PFD, pulmonary fibrosis as a diagnostic label; TLCO, transfer factor for carbon monoxide.

Due to high levels of data missingness for FVC, TLCO and oxygen (L/min) at admission (online supplemental table 3), an assessment of missingness was undertaken. Logistic regression was used to compare data missingness for FVC, TLCO and oxygen (L/min) at admission with age, CCI, deprivation decile and 90-day mortality outcomes. Associations were observed (online supplemental table 4) suggesting missingness may be consistent with a missing-at-random (MAR) mechanism. Subsequent multiple imputation modelling for FVC, TLCO and oxygen (L/min) at admission was undertaken. Multiple imputation using chained equations (MICE) generated 20 imputed datasets. Updated Cox proportional hazards models using the ENTER method were fitted across the imputed datasets and pooled using Rubin’s rules.^11^ The imputation model included all variables used in the complete case analysis model. The pooled results are reported. A comparison of HRs output between complete case analysis and pooled analysis was also undertaken.

Finally, due to significant age differences observed between quintiles in both initial Kruskal-Wallis H analysis (table 1) and in post-hoc pairwise comparison (online supplemental table 1), sub-analysis to understand the interaction between age and deprivation was undertaken. An interaction analysis using an age × deprivation term was completed on the pooled analysis model. A p value of <0.05 was considered statistically significant.

## Results

3451 admissions were identified through primary ICD-10 codes relevant to the study. 2452/3451 (71.05%) were excluded, with the predominant reason being attendance at day case procedures (1984/2449, online supplemental figure 1). Other reasons for exclusion included transplant work-up attendances (158/2449), inability to access medical record relevant to admission (118/2449), non-ILD related admission (118/2449) and incorrect coding (67/2449, online supplemental figure 1). Three admissions were excluded due to absence of postcode to calculate deprivation score, leaving 999 records included in data analysis. Quintile 5, representing the most deprived 20% (DDs 1 and 2), accounted for 327/999 (32.7%) of admission events.

### Baseline characteristics

Complete descriptions of the baseline characteristics for each quintile cohort are described in table 1. Several variables showed statistically significant differences at initial data interrogation: age, ethnicity, CCI, smoking status, ILD subtype, antifibrotic use and AEILD status.

Post-hoc analysis supported greater understanding. Pairwise comparison of age demonstrated that quintile 5 was the main outlier, with a statistically significant younger cohort on average when compared with quintile 1 (p=0.0006), quintile 2 (p<0.0001) and quintile 4 (p=0.003, online supplemental table 1). The difference in co-morbidity burden (as per CCI values) was observed between quintiles 2 and 5 (p=0.015), and quintiles 4 and 5 (p=0.003), in post-hoc pairwise comparison—with quintile 5 demonstrating the lowest co-morbidity burden using this index (online supplemental table 1).

Categorical variables underwent post-hoc analysis interrogation using standardised residuals. Full results of residuals, raw p values and Bonferroni correct p values are available in online supplemental table 2. During an initial assessment, smoking status demonstrated a statistically significant χ² value; however, this effect was not demonstrated in post-hoc analysis. Similar patterns were observed for ILD subtype, antifibrotic use and ILD status. Of note however, prior to Bonferroni correction, analysis suggested a trend to increased numbers of NSIP sub-type within quintile 1 (raw p value=0.007, (online supplemental table 2), vs reduced numbers of NSIP within quintile 2 (raw p value=0.050, (online supplemental table 2)—but statistical significance was not observed with correction. Quintile 5 demonstrated a pattern of more patients with an ‘other’ ILD subtype (raw p value=0.002, online supplemental table 2), inclusive of several ILD subtypes, but this did not remain significant at the point of Bonferroni correction.

### Primary outcome: 90-day all-cause mortality

**Figure 1:**
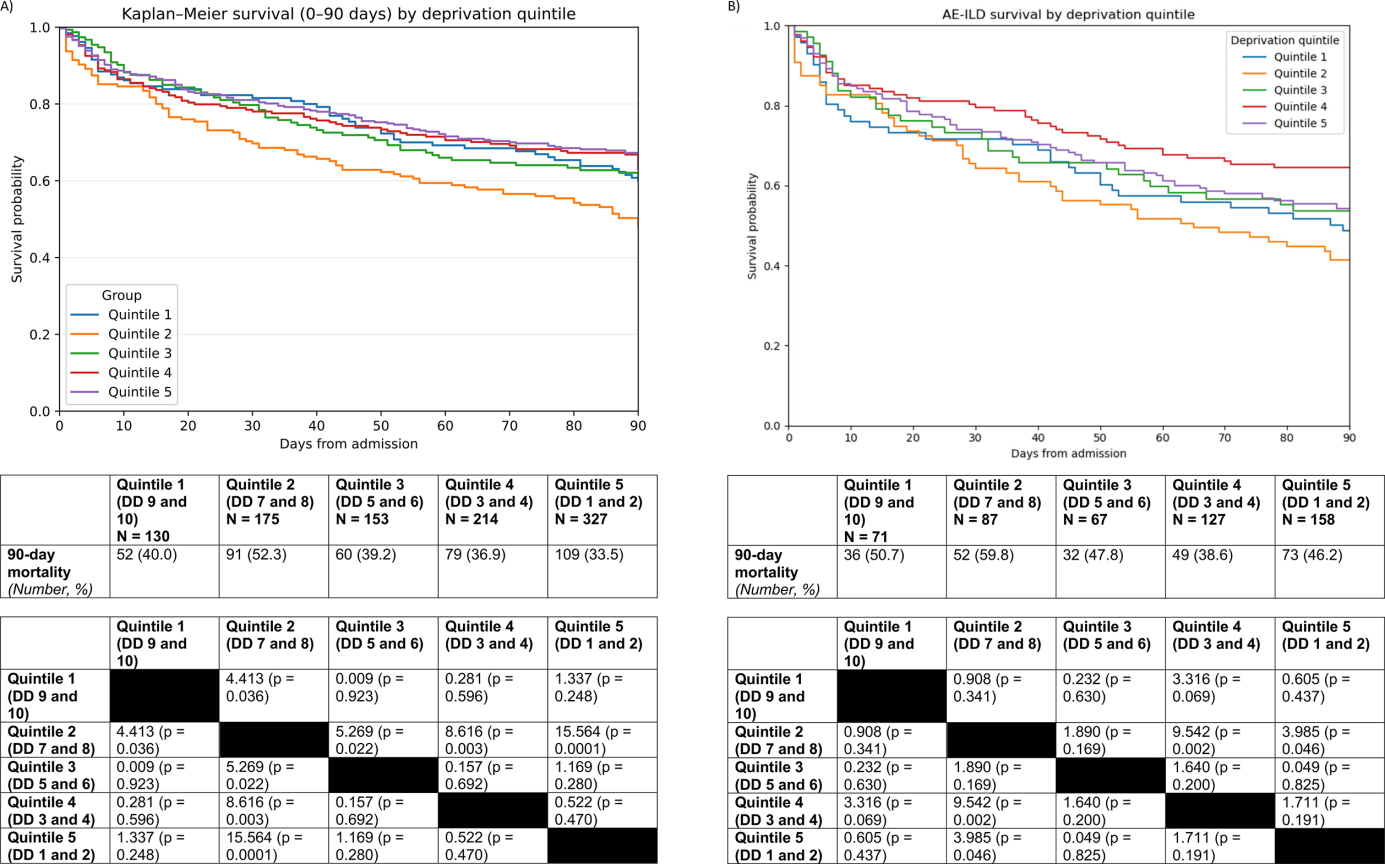
(A) Kaplan-Meier time-to-event analysis of 90-day all-cause mortality comparing deprivation quintiles across all acute interstitial lung disease (ILD)-related hospital admissions. (B) Kaplan-Meier time-to-event analysis of 90-day all-cause mortality comparing deprivation quintiles for acute exacerbation of interstitial lung disease (AEILD) subgroup, encompassing both suspected (not confirmed with CT imaging) and definite (confirmed with CT imaging) AEILD events. Quintile 1 represents the least deprived 20% and quintile 5 represents the most deprived 20%. The accompanying tables report 90-day all-cause mortality outcomes as number of events (death) and as a percentage of all acute ILD-related admissions within the relevant deprivation quintile, alongside log-rank pairwise comparisons of 90-day all-cause mortality between quintile groups. A p value of <0.05 was considered statistically significant. DD, deprivation decile.

The overall 90-day mortality across all quintiles was 39.1%. Time-to-event analysis demonstrated an overall statistically significant difference in 90-day all-cause mortality between the deprivation quintiles (p<0.002, figure 1a) with subsequent pairwise comparison revealing significant differences in survival between quintile 2 and all other quintiles (2 vs 1 p=0.036; 2 vs 3 p=0.022; 2 vs 4 p=0.003; 2 vs 5 p=0.0001, figure 1).

Secondary outcomes: 90-day all-cause mortality in AEILD subgroup

510/999 admissions met AEILD criteria, encompassing suspected AEILD (without CT confirmation) and definite AEILD (with CT confirmation). The overall 90-day mortality across all quintiles in the AEILD sub-group was 47.5%. Simple modelling with time-to-event analysis and pairwise comparison revealed significant difference in all-cause 90-day mortality between quintiles 2 and 4 (p=0.002, figure 1) and quintiles 2 and 5 (p=0.046, figure 1).

### Secondary outcomes: multivariate modelling of 90-day all-cause mortality

11 variables were included in a multivariate model for 90-day all-cause mortality with complete case analysis (table 2). Prior to completing, the proportional hazards assumption was checked. There was no evidence of widespread violation. Minor violations were suggested for CCI and one ILD subtype. Key variables associated with increased risk of mortality in this model included male gender (HR 1.343, 95% CI 1.070 to 1.687, p=0.011; table 2), long-term oxygen therapy (HR 1.931, 95% CI 1.528 to 2.440, p = <0.0001, table 2) and increased neutrophil count (HR 1.083, 95% CI 1.052 to 1.115, p<0.0001, table 2). A higher deprivation decile (representing lower deprivation) showed a borderline association with increased mortality (HR of 1.038, 95% CI 1.000 to 1.077, p=0.050).

**Table 2:** Full results of multivariate Cox regression analysis of 90-day all-cause mortality associated with acute interstitial lung disease-related hospital admissions, using complete case analysis

	HR	Lower 95% CI	Upper 95% CI	P value
Age	1.027	1.013	1.041	**0.0002**
Sex Female Male	Reference 1.343	Reference 1.070	Reference 1.687	Reference 0.011*
Ethnicity White Asian Black Not stated	Reference 0.636 1.739 1.537	Reference 0.389 0.511 1.013	Reference 1.039 5.920 2.332	Reference 0.071 0.376 0.043*
CCI	1.012	0.941	1.087	0.752
Deprivation decile	1.038	1.000	1.077	**0.050**
ILD subtype Not stated IPF NSIP CTD-ILD HP Drug-related Industry-related Sarcoidosis PFD Unclassifiable Other	Reference 1.136 0.547 1.260 0.652 0.256 0.781 0.542 1.028 0.883 1.310	Reference 0.722 0.203 0.739 0.329 0.121 0.295 0.185 0.425 0.549 0.446	Reference 1.785 1.473 2.146 1.295 0.545 2.070 1.587 2.491 1.420 2.625	Reference 0.582 0.232 0.396 0.222 **0.0004** 0.620 0.264 0.950 0.607 0.654
Oxygen None Long-term Ambulatory	Reference 1.931 1.358	Reference 1.528 0.922	Reference 2.440 1.999	Reference **<0.0001** 0.121
AEILD status Insufficient information AEILD Other ILD-related admission	Reference 1.325 0.904	Reference 0.785 0.527	Reference 2.234 1.550	Reference 0.292 0.713
Neutrophils	1.083	1.052	1.115	**<0.0001**
Monocytes	0.876	0.682	1.126	0.301
CRP	1.002	1.001	1.004	**0.004**

* Statistically significant values are marked in bold.

AEILD, acute exacerbation of interstitial lung disease; CCI, Charlson Comorbidity Index; CRP, C-reactive protein; CTD-ILD, connective tissue disease interstitial lung disease; HP, hypersensitivity pneumonitis; ILD, interstitial lung disease; IPF, idiopathic pulmonary fibrosis; NSIP, non-specific interstitial pneumonia; PFD, pulmonary fibrosis as a diagnostic label.

Given the high level of missing data for potentially key variables (FVC, TLCO and oxygen (L/min) at admission), repeat analysis using pooled data from MICE was undertaken (table 3). Of note, male gender (HR 1.518, p<0.0001, table 3) and long-term oxygen therapy (HR 2.770, p<0.0001, table 3) remained strongly associated with increased risk of mortality. Lower TLCO values were significantly associated with increased risk of mortality in this model (HR 0.890, 95% CI 0.841 to 0.941, p<0.0001, table 3). A direct comparison of the models is available in table 4.

**Table 3:** Full results of multivariate Cox regression analysis of 90-day all-cause mortality associated with acute interstitial lung disease-related hospital admissions, using multiple imputation modelling for FVC, TLCO and oxygen required at admission (litres)

	HR	Lower 95% CI	Upper 95% CI	P value
**Age**	1.016	1.005	1.027	**0.003**
Sex Female Male	Reference 1.518	Reference 1.244	Reference 1.852	Reference <0.0001*
Ethnicity White Asian Black Not stated	Reference 0.914 1.060 0.842	Reference 0.647 0.453 0.581	Reference 1.292 2.478 1.220	Reference 0.611 0.893 0.364
**CCI**	1.032	0.965	1.104	0.354
Deprivation decile	1.001	0.971	1.033	0.924
ILD subtype Not stated IPF NSIP CTD-ILD HP Drug-related Industry-related Sarcoidosis PFD Unclassifiable Other	Reference 1.285 1.050 0.999 0.948 0.634 1.180 1.042 1.597 1.062 1.245	Reference 0.921 0.698 0.654 0.653 0.360 0.617 0.604 1.164 0.578 0.771	Reference 1.794 1.578 1.527 1.376 1.118 2.255 1.800 2.193 1.952 2.001	Reference 0.140 0.816 0.998 0.779 0.116 0.617 0.883 0.004* 0.846 0.369
Oxygen None Long-term Ambulatory	Reference 2.770 2.020	Reference 2.113 1.448	Reference 3.633 2.818	Reference <0.0001* <0.0001*
AEILD status Insufficient information AEILD Other ILD-related admission	Reference 1.340 1.472	Reference 0.951 1.060	Reference 1.890 2.046	Reference 0.094 0.021*
Neutrophils	1.028	0.995	1.062	0.092
Monocytes	1.063	0.980	1.153	0.143
CRP	0.998	0.996	0.999	0.028*
FVC (L)	1.001	0.991	1.022	0.405
TLCO (mm Hg)	0.890	0.841	0.941	<0.0001*
Oxygen (L/min) at admission	0.979	0.936	1.024	0.349

* Statistically significant values are marked in bold.

AEILD, acute exacerbation of interstitial lung disease; CCI, Charlson Comorbidity Index; CRP, C-reactive protein; CTD-ILD, connective tissue disease interstitial lung disease; FVC, forced vital capacity; HP, hypersensitivity pneumonitis; ILD, interstitial lung disease; IPF, idiopathic pulmonary fibrosis; L, litres; L/min, litres per minute; NSIP, non-specific interstitial pneumonia; PFD, pulmonary fibrosis as a diagnostic label; TLCO, transfer factor of the lung for carbon monoxide.

**Table 4:** Direct comparison of HR values between complete case and multiple imputation Cox regression modelling

	HR—complete case model	P value	HR—pooled model	P value
Age	1.027	**0.0002**	1.027	**0.003**
Sex Female Male	Reference 1.343	Reference 0.011*	Reference 1.518	Reference <0.0001*
Ethnicity White Asian Black Not stated	Reference 0.636 1.739 1.537	Reference 0.071 0.376 0.043*	Reference 0.914 1.060 0.842	Reference 0.611 0.893 0.364
CCI	1.012	0.752	1.032	0.354
Deprivation decile	1.038	0.050*	1.001	0.924
ILD subtype Not stated IPF NSIP CTD-ILD HP Drug-related Industry-related Sarcoidosis PFD Unclassifiable Other	Reference 1.136 0.547 1.260 0.652 0.256 0.781 0.542 1.028 0.883 1.310	Reference 0.582 0.232 0.396 0.222 **0.0004** 0.620 0.264 0.950 0.607 0.654	Reference 1.285 1.050 0.999 0.948 0.634 1.180 1.042 1.597 1.062 1.245	Reference 0.140 0.816 0.998 0.779 0.116 0.617 0.883 0.004* 0.846 0.369
Oxygen None Long-term Ambulatory	Reference 1.931 1.358	Reference <0.0001* 0.121	Reference 2.770 2.020	Reference <0.0001* <0.0001*
AEILD status Insufficient information AEILD Other ILD-related admission	Reference 1.325 0.904	Reference 0.292 0.713	Reference 1.340 1.472	Reference 0.094 0.021*
Neutrophils	1.083	<0.0001*	1.028	0.092
Monocytes	0.876	0.301	1.063	0.143
CRP	1.002	0.004*	0.998	0.028*
FVC (L)	N/A	N/A	1.001	0.405
TLCO (mm Hg)	N/A	N/A	0.890	<0.0001*
Oxygen (L/min) at admission	N/A	N/A	0.979	0.349

* Statistically significant values are marked in bold.

AEILD, acute exacerbation of interstitial lung disease; CCI, Charlson Comorbidity Index; CRP, C-reactive protein; CTD-ILD, connective tissue disease interstitial lung disease; FVC, forced vital capacity; HP, hypersensitivity pneumonitis; ILD, interstitial lung disease; IPF, idiopathic pulmonary fibrosis; L, litres; L/min, litres per minute; N/A, Not applicable; NSIP, non-specific interstitial pneumonia; PFD, pulmonary fibrosis as a diagnostic label; TLCO, transfer factor of the lung for carbon monoxide.

Given the significant age difference observed, multivariate modelling with the pooled data was re-run with an age × deprivation term. In this model, age × deprivation was not significantly associated with increased or reduced risk of mortality (HR 0.999, 95% CI 0.996 to 1.001, p=0.258, online supplemental table 5).

## Discussion

### Summary of results

This retrospective real-world dataset infers a potentially complex, multifactorial association between social deprivation and 90-day all-cause mortality outcomes from acute ILD-related admissions. Of note, we observe high rates of admission among the geographically most deprived 20%—with 32.7% (327/999) of acute ILD-related admissions from this group.

When considering mortality outcomes, unadjusted survival analysis demonstrates a non-linear relationship between mortality outcomes and social deprivation—with the key difference observed in pairwise comparison between quintile 2 and quintile 5. 90-day all-cause mortality in quintile 2 was 52.3% vs 33.5% in quintile 5 (p=0.0001, figure 1). In a complete case multivariate model with eleven variables, lower deprivation demonstrates a borderline increased risk of mortality (HR 1.038, 95% CI 1.000 to 1.077, p=0.050, table 2). However, this borderline significance is lost in pooled analysis where values for FVC, TLCO and oxygen (L/min) at admission are imputed (HR 1.001, 95% CI 0.971 to 1.033, p=0.924, table 3). In such multivariate modelling, male gender and pre-admission long-term oxygen therapy remain consistently statistically significantly associated with increased mortality (table 4). In pooled analysis modelling, the significant association of lower TLCO values with increased mortality is demonstrated (HR 0.890, p<0.0001, tables 3 and 4).

AEILD sub-group analysis demonstrated a similar non-linear relationship in unadjusted survival analysis. We observed statistically significant differences in survival between quintiles 2 and 4 (90-day mortality 59.8% vs 38.6%, p=0.002), and 2 and 5 (90-day mortality 59.8% vs 46.2%, p=0.046, figure 1). Further multivariate modelling was not undertaken due to lack of power but should be explored in future studies.

When comparing across quintiles, significant differences in baseline characteristics were noted. Of particular interest was age, with quintile 5 (the most deprived 20%) demonstrating a statistically significantly reduced age compared with other quintiles, even in post-hoc pairwise comparisons: quintile 1 vs 5 p=0.0006; quintile 2 vs 5 p<0.0001; quintile 3 vs 5 p=0.161; quintile 4 vs 5 p=0.003 (table 1 and online supplemental table 1). Given this, we assessed for the potential of age differences contributing to the pattern observed above. In multivariate modelling, using pooled analysis, an age × deprivation term did not show statistically significant association with mortality (HR 0.999, 95% CI 0.996 to 1.001, p=0.258, online supplemental table 5). Including this term did increase the numerical HR observed for deprivation in multivariate modelling (HR 1.110) but this did not meet statistically significant thresholds (p=0.256, online supplemental table 5).

### Study strengths and limitations

A key strength of this study is its multicentre design, encompassing secondary and tertiary hospitals from across the North West of England region. However, there are several important limitations to consider.

This study timeframe (2017–2019) was chosen to remove COVID-19 as a potential confounder, given its impact among higher deprivation populations.^12^ But, we recognise the treatment landscape of ILD has changed significantly since the 2017–2019 period. This is particularly pertinent for the use of antifibrotics—which became widespread following the results of the Inbuild study^13^ and change in UK National Institute for Health and Care Excellence guidelines. There have also been significant changes to care pathways in UK ILD services which may impact the results we have observed.^14^ Given the small numbers of patients receiving antifibrotics within our cohort, we did not include this variable in our modelling. This is a significant limitation which may impact the relevance of its findings to current populations. Future studies should seek to gather data prospectively to build an accurate picture of the impact of antifibrotics on outcomes—especially within the context of deprivation.

Retrospective data risks recall and misclassification bias—and there were significant challenges with missing data in this dataset (online supplemental table 2). As a result, MICE was required to infer the impact of FVC, TLCO and oxygen (L/min) at admission on 90-day mortality outcomes. It also meant we were unable to reliably include the GAP index in our multivariate modelling. Given this, our results may not reflect true outcomes and future studies should aim to gather data prospectively so that more complete datasets can be achieved. Additionally, there is the potential for misclassification bias given AEILD status was based on the opinion of the site-specific data collector. Inter-rate reliability was not assessed, and thus consistency in diagnosis may be impacted. Admission coding adds to this challenge. Given the potential for incorrect coding, it is unlikely all cases from across this period have been identified—which may have impacted the deprivation skew we observed.

A further limitation is the baseline deprivation within the North West of England population. We recognise that this region has, comparatively, high levels of deprivation and as such our dataset is skewed to including patients from areas of high deprivation. This may limit the generalisability of our findings. However, geographical deprivation does not always equate to individual-level deprivation. In future prospective studies, researchers should look to include more detailed individual-level deprivation markers.

### Findings in context

Our findings are contrary to previous observations in chronic forms of ILD^5^—raising important questions about why this has been observed. To the best of our knowledge, this is the first time social deprivation has been studied in the setting of acute ILD-related admissions. Acute hospitalisations, including acute exacerbations, are well-recognised to be significant events in the trajectory of ILD.^15 16^ However, our findings suggest a potentially complex, multifactorial association between social deprivation and 90-day all-cause mortality in acute ILD-related admissions.

Our unadjusted survival analysis revealed a non-linear relationship between deprivation and mortality outcomes, with those experiencing low-intermediate deprivation (DDs 7 and 8, quintile 2) demonstrating a significantly worse survival compared with many other deprivation quintiles (figure 1). There are several potential explanations for this. One is the difference in age and comorbidity burden, particularly between quintiles 2 and 5, which remains significant in post-hoc analysis (online supplemental table 1). In other conditions, such as community-acquired pneumonia, age and comorbidities can interact to worsen mortality outcomes^17^—and thus there is potential these factors play significant roles. However, in multivariate modelling, we did not observe age or comorbidities (reported as CCI) as significant factors associated with mortality (tables 2–4)—even when a specific age × deprivation term is developed (online supplemental table 5).

Other potential explanations for this non-linear relationship may include more advanced pre-existing ILD prior to the admission event. Differences in TLCO, especially disproportionately lower TLCOs, may infer co-existing pulmonary arterial hypertension (PAH). Co-existing PAH has previously been associated with poorer mortality outcomes.^18^ Such patterns of disease severity in our cohort may occur due to chance, or due to true differences between the cohorts. Using available data, we did not observe a statistically significant difference across the quintiles in mean FVC values or mean TLCO values (table 1). This must be interpreted with high caution given the high rates of missing data—meaning absolute conclusions cannot be drawn. Prospective studies should be undertaken to re-assess this possibility, especially given the strong signal in multivariate modelling suggesting lower TLCO values may be associated with mortality outcomes (table 3), consistent with prior findings.^19 20^

A final explanation for these findings is that DD is not representative of true individual-level deprivation. The UK 2019 English Indices of deprivation use averages of seven deprivation factors within an area, resulting in postcodes being representative of geographical level deprivation. IMDs have been shown to be poor proxies for individual income, especially among groups with poor health.^21^ In COVID-19, geographical deprivation as a marker of individual deprivation may have resulted in inaccurate assessments.^22^ As such, our marker of deprivation may not reflect the true individual-level deprivation score—resulting in an over-estimation or under-estimation of deprivation and skewing of our quintiles subsequently. Future studies should look to include additional markers of individual deprivation, such as dental health,^23^ educational attainment and air pollution.^24^

As such, we hypothesise the non-linear relationship observed in unadjusted analysis may be secondary to multiple, likely interacting factors, that are not fully explained in our dataset. Future studies are needed to first understand if this pattern is observed in repeat datasets.

### Admission patterns: the impact of social deprivation

A key finding of our retrospective dataset is the high admission burden from the most deprived 20%. It is well recognised that emergency attendances and admission rates are higher among the most deprived,^25^ reflecting the challenges of health literacy and accessing community services and support.^26^ Prior research has shown that those from higher deprivation have significantly reduced healthy life years.^27^ Reduced healthy life years are likely as a result of combined individual and geographical deprivation factors. At an individual level, poor diet,^28^ poor housing^29^ and an inability to afford travel to primary, secondary and tertiary care appointments.^30^ There is also a suggestion of a lack of trust in public institutions, including the NHS, from the most deprived groups^31^—impacting their decision to access healthcare further. At a geographical level, there are fewer GPs per patient in more deprived areas and greater waits for non-urgent care.^32 33^

Our findings are suggestive of these challenges in our ILD population which raises important considerations for future policy, systems and research. For research, we need to understand the geographical and individual-level deprivation factors in more detail so that we can design a service that supports this group of patients. While this has been assessed more generally, revealing many layers of reason for delayed or challenging healthcare access,^30^ there may be specific challenges unaccounted for in ILD care given the significant unmet needs many in the service experience.^34^ As a way to begin addressing these issues, we need to increase awareness and education of ILDs among patients, caregivers and primary care physicians working in such deprived areas. By going out into communities, into settings that such patients frequent, we can build this awareness but also trust. Policymakers must remain acutely aware of such groups in future service planning, especially in the context of rapid digital healthcare expansion, so that new services or interventions in ILD do not inversely worsen inequalities for this group of patients.^35^

## Conclusion

From our dataset, we can cautiously infer that social deprivation impacts on health-seeking behaviours—with higher demands on acute healthcare services. However, once admitted in the context of an acute ILD-related admission, the association between deprivation and mortality outcomes becomes much more complex and multifactorial—and the impact of geographical deprivation reduced. Further studies are needed to examine this association in more detail, with careful consideration of geographical vs individual-level markers of deprivation.

## Supplementary material

10.1136/bmjresp-2025-003944 online supplemental file 1

## Data Availability

Data are available upon reasonable request.
